# Community-Level Incentive Mechanisms for the Conservation of Crop Wild Relatives: A Malawi Case Study

**DOI:** 10.3390/plants12051030

**Published:** 2023-02-24

**Authors:** Adam G. Drucker, Nolipher Khaki Mponya, Francesca Grazioli, Nigel Maxted, Joana Magos Brehm, Ehsan Dulloo

**Affiliations:** 1 Bioversity International, Via di San Domenico 1, 00153 Rome, Italy; 2 Malawi Plant Genetic Resources Centre (MPGRC), Chitedze Research Station, Lilongwe P.O. Box 158, Malawi; 3 School of Biosciences, University of Birmingham, Birmingham B15 2TT, UK

**Keywords:** crop wild relatives, ecosystem services, community engagement, conservation incentives, payments for agrobiodiversity conservation services

## Abstract

Despite being an increasingly important source of genes for crop breeding aimed at improving food security and climate change adaptation, crop wild relatives (CWRs) are globally threatened. A root cause of CWR conservation challenges is a lack of institutions and payment mechanisms by which the beneficiaries of CWR conservation services (such as breeders) could compensate those who can supply them. Given that CWR conservation generates important public good values, for the significant proportion of CWRs found outside of protected areas, there is a strong justification for the design of incentive mechanisms to support landowners whose management practices positively contribute to CWR conservation. This paper contributes to facilitating an improved understanding of the costs of in situ CWR conservation incentive mechanisms, based on a case study application of payments for agrobiodiversity conservation services across 13 community groups in three districts in Malawi. Results demonstrate a high willingness to participate in conservation activities, with average conservation tender bids per community group being a modest MWK 20,000 (USD 25) p.a. and covering 22 species of CWRs across 17 related crops. As such, there appears to be significant potential for community engagement in CWR conservation activities that is complementary to that required in protected areas and can be achieved at modest cost where appropriate incentive mechanisms can be implemented.

## 1. Introduction

Crop wild relatives (CWRs) are wild plant taxa, that include the progenitors of crops, as well as taxa more or less closely related to crops [1,2]. CWRs are a diverse group of plants, occurring in a wide variety of habitats and all continents (except Antarctica). Globally, it is estimated there are 50,000 to 60,000 CWR species, although only about 11,000 of these are estimated to be of direct value to food security, which is most closely related to the most important crops [3]. A total of 1392 CWR species related to 173 crops are considered as a priority globally [4]. CWRs constitute an increasingly important source of genetic diversity for breeding. Identification and transfer of useful traits from 185 CWR taxa into 29 crop species has been reported [3], reflecting the recognition that crop improvement strategies rely on a continued supply of genetic diversity and associated beneficial traits, of which CWRs are a major source [5,6]. They provide cultivars with pest and disease resistance, heat and drought tolerance, tolerance of salinity and other abiotic stresses, and enhanced nutritional quality and yield [3,6,7,8]. It is estimated that approximately 30% of modern crop production increase is due to the use of CWR genetic diversity and that this has an annual value of approximately USD 115 billion worldwide [9]; while more recently it has been estimated CWRs contribute USD 120 billion to increased crop productivity per annum considering just 29 of the world’s priority crops [10].

Despite their importance, CWRs are globally threatened with extinction and the genetic erosion of their innate diversity due to agricultural intensification, habitat destruction, and a range of other threats including land-use change [11,12]. Efforts to improve conservation are therefore warranted to reduce further loss of diversity [2,13]. While a complementary approach to conservation (i.e., involving application of in situ and ex situ techniques) is desirable, in practice, the principal strategy used to date has been to collect and store seed from CWRs in genebanks [2,13]. However, in recent times, there has been an increased impetus by national governments to promote the in situ conservation of CWRs [14].

To avoid placing all our conservation “eggs in a single basket” and readdressing the CWR complementary conservation challenge, institutions and payment mechanisms by which the beneficiaries of CWR conservation services could compensate those who can supply them are urgently needed [15]. Such a situation arises given that direct users of CWR diversity (such as breeders) are generally distant from where CWRs are found in nature. Furthermore, CWR conservation generates important public good values (*inter alia*, contributing to global food security, resilient landscapes and the maintenance of future ecosystem option values). For those CWRs found outside of protected areas and threatened by agricultural intensification, there is thus a strong justification for the design of incentive mechanisms to support landowners whose management practices positively contribute to CWR conservation. 

Attempting to secure a strategic conservation portfolio of plant genetic resources through the development of niche product markets alone has been shown to have limited potential [16]. For those genetic resources without direct market potential, agri-environmental schemes (AES), and payments for ecosystem services (PES) approaches—which have been widely applied as incentive mechanisms to motivate natural resource conservation, where important public good ecosystem service values exist (see [17,18,19] for recent overviews), —have been developed for specific application to agrobiodiversity conservation [20,21,22]. 

However, the implementation of PES-type schemes for agrobiodiversity conservation has been limited [20,21], especially with regard to CWR (although see [23] for a conceptual application in Zambia; and [24] for a report of actual implementation in the UK). As a result, the costs of CWR conservation often remain largely unknown at both programme and community levels, thereby limiting the ability to assess the viability of in situ approaches (although for exceptions, see [25] for back of envelope calculations, as well as [23]). 

Such payments for agrobiodiversity conservation services (PACS) have been implemented for a range of crops including quinoa in Peru and Bolivia, amaranth and potato in Peru, maize in Ecuador, and beans and maize in Guatemala (see [21] for an overview). Hypothetical applications have also been undertaken in India [26] and Nepal [27] for minor millets, as well as specifically for CWRs in Zambia [23]. Under PACS, farmers are rewarded for conserving threatened genetic resources of high public good value. In developing country contexts, incentives have been offered at community level and involve landscape-wide competitive tender (*spatial targeting concept*). Groups define their participation conditions (i.e., which priority species/varieties to cultivate from a given portfolio, kind and level of reward needed, which farmers participate). *Efficiency and social equity criteria* (including gender) are used to select communities with the most attractive bids (*payment differentiation concept*). PACS competitive tenders are a reverse auction mechanism, whereby farmers submit a bid offer for a pre-defined conservation contract supplying, in this instance, CWR conservation services. Relative to fixed price approaches, competitive tenders are incentive compatible, allowing farmers to reveal their true opportunity costs (which include both market and non-market values and preferences). This allows the identification of least-cost suppliers, which when combined with criteria related to gender, youth and poverty, allow for the design of cost-effective, socially-just conservation programmes [22]. 

Once communities have been contracted, compliance verification/monitoring (*conditionality concept*, *effectiveness criteria*) visits are carried out at certain key moments during the agricultural season and reward handover ceremonies realised upon successful completion of contracts (see [21] (p. 3) for an illustration of the different steps involved and their timing). In developed country contexts, more individual approaches and the use of cash payments may be considered appropriate [24].

There is therefore a continued need to explore innovative incentive mechanisms in the context of CWR to both motivate community-based conservation activities, as well as to facilitate a fair and equitable sharing of the benefits of genetic resources conservation and use (as per calls under the Global Biodiversity Framework [Targets 13 and 17] and the Nagoya Protocol of the Convention on Biological Diversity, as well as the International Treaty on Plant Genetic Resources for Food and Agriculture) with a view to facilitating an improved understanding of the costs of in situ CWR conservation. This paper addresses this need, and the remainder of this paper is structured as follows. Section 2 describes the Malawian research context, sites, and the methodological and modelling approach used. Section 3 presents the results and a discussion of the PACS tender outcomes, while Section 4 presents conclusions and recommendations for further applied research.

## 2. Materials and Methods

### 2.1. Study Sites

Malawi is landlocked, located in southern central Africa and is one of the poorest countries in the world. It is ranked 169 out of 191 countries in the UN Human Development Index [28], with an average per capita income per annum in 2021 of USD 635 or USD 1.74 per day [29]. The country is divided into three regions: the undulating and densely populated Southern Region; the well-populated Central Region with its fertile plains; and the mountainous, sparsely populated Northern Region. Of the 9.4 million hectares of land available for agriculture, approximately 32% are suitable for rainfed agriculture. Agriculture is the mainstay of Malawi’s economy, contributing approximately 31% of GDP. Overall, 83% of households in Malawi are engaged in agricultural activities. However, the agricultural sector is dualistic. Estate agriculture accounts for more than 25% of agricultural GDP and 90% of export earnings. The main crops are tobacco (60%) tea (20%), and sugar (18%). By contrast, 3.1 million smallholder farmers can be found on 6.5 million hectares of land—69% of Malawi’s total land area. A total of 78% of such households cultivate land during the rainy season while only 8% of households practice dry season crop production. Average cultivated area per household is 0.61 ha. with approximately 60% of smallholder farmers cultivating less than 1.0 ha of land; 67%t of plots are intercropped with two crops, while 23% are intercropped with three crops and 5% with four crops. The main crops are maize (76% of plots), pigeon pea (19.3%), groundnuts (10.2%), beans (9.8%), soya (6.3%), tobacco (4.2%), and rice (3%) [30,31]. 

**Figure 1:**
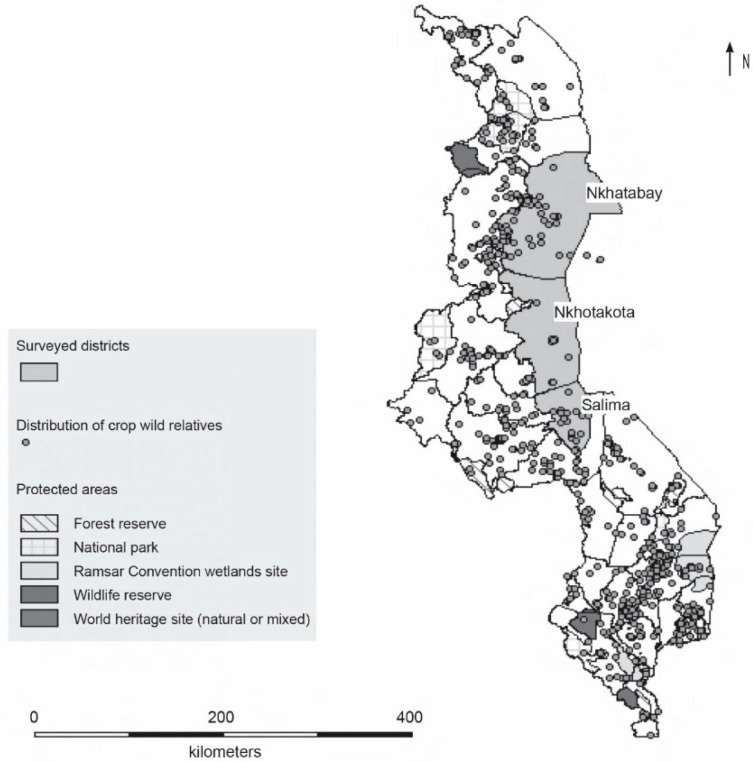
Project sites and distribution of crop wild relatives in Malawi.

Malawi has 446 crop wild relatives, with 74.7% of the taxa being native to Malawi. In general, these CWR are distributed across the country, with approximately 73% being found in protected areas [32] (see Figure 1 and Appendix A). Coverage outside the protected areas (PAs) is concentrated in agricultural areas across 10 districts (Chikwawa, Dedza, Dowa, Nkhatabay, Nkhotakota, Ntchewu, Nsanje, Ntchisi, Lilongwe, and Salima). Surveys were undertaken in the districts of Nkhatabay, Nkhotakota and Salima (as shown in Figure 1). Nkhatabay is located in the Northern Region of Malawi at an elevation of 472 metres above sea level (latitude −11.608556 and longitude 34.294941). The district receives heavy rains and has the longest wet period (7.2 months per year) with January being the wettest month (568.42 mm) with an average monthly precipitation of 472.86 mm. A large proportion of the district is under forest cover dominated by evergreen trees, unlike Salima and Nkotakota where most of the area is grassland. Nkhotakota is in the Central Region of Malawi located along the lakeshore of Lake Malawi at an elevation of about 476 metres above sea level (latitude −12.931686 and longitude 34.281055). The district experiences hot to humid weather most of the year with average rainfall of about 1523 mm. The wet period lasts for about four months with the dry period starting from mid-April and ending in November. Salima district is located in the Central Region of Malawi along the shores of Lake Malawi. Salima has an altitude of 538 metres above sea level (latitude −13.78074000 and longitude 34.45848100). The district experiences dry and hot weather throughout the year with 405 mm average rainfall per annum. 

The survey sites were selected based on previous ecogeographic surveys that indicated the distribution of CWRs (as indicated by the dots in Figure 1) in these sites [32], as well as from information obtained during consultations with key informants. In Nkhatabay the surveys were conducted in the Chitheka Extension Planning Area (EPA) on the outskirts of the South Viphya Forest Reserve. In Nkhotakota, the surveys were conducted in Mphonde EPA (Buamufu, Lunga 1 and Ngalatete Sections) and Linga EPA (Chilingali, Kasamba West, Ling’ona, and Sasani Sections); while in Salima, the survey was conducted in Chipoka EPA (Lifidzi Section) and Tembwe EPA (Maganga Section). 

### 2.2. Methods

With a view to implementing PACS specifically in the context of CWRs (as opposed to crop varieties *per se*) and building on a previous PACS CWR conceptual application in Zambia [23], a range of survey protocols were developed and applied. Following the identification of the priority locations for CWR conservation, focus group discussions (FGDs) were held with communities in these areas, transect walks were realised to verify the presence of CWRs as reported by community members, and conservation tenders (CT) were realised. Following the selection of the CT bid offers, conservation activities were initiated by the community groups. Monitoring, verification, and capacity building visits were undertaken by project personnel, including regarding supporting post-project sustainability planning. Upon successful completion of the conservation activities at the end of the project, reward handover ceremonies were realised (see Table 1 for an example of the different steps and their timing).

#### 2.2.1. Focus Group Discussions

The focus group discussions (FGDs) sought to identify CWR diversity, population status, and trends, as well as adaptive traits; understand the degree of recognition of CWR within communities and their management practices (if any); as well as to discuss and identify the specific tasks (and associated costs, as perceived by the community members) that would need to be implemented in order to attain a desirable level of conservation management. Each FGD lasted 2–3 h with 15–25 participants in each, with the aim of encompassing a mix of genders and age groups—i.e., youths/younger farmers (15–25 years old), middle-aged farmers (25–45 years old), and older farmers (>45 years old). For large groups of farmers (20 or more), the group was divided into two. The FGD participants were identified and randomly selected by Agriculture Extension Development Officers across the EPAs. Each FGD was comprised of participants from 2–3 communities, which in the surveyed areas ranged in size from 35–50 households. Malawi National Plant Genetic Resource Centre gene bank staff organised and conducted the FGDs.

Before conducting the FGDs, consent to participate was obtained from the participants along with the management of personal data, in accordance with institutional guidelines and approval. Flip charts, markers, iPads, and cameras were used to record the information during the discussions. 

Prior to initiating a semi-structured, open-ended discussion, the FGD facilitators ensured that there was a common understanding of what crops wild relatives are, their importance in breeding programmes, and the role they play in maintaining ecosystems services of importance to human livelihoods. A checklist of questions was used to capture information from farmers relating to their knowledge of the CWRs present within their communities. Farmers were first asked to list all the CWRs known to be present in their communities and were subsequently requested to relate these to a list of all the crops cultivated in their area. Once the listing of CWRs was complete, farmers were then asked to, *inter alia*, detail any direct use or benefits that accrued to them associated with the listed CWRs, as well as to describe the sites where they were found, population trends, and reasons for changes, management practices (if any), and associated costs. Participants were also asked to bring in samples of the CWRs occurring in their communities and participant–facilitator transect walks were realised to validate that the stated CWRs were actually present in their communities (as per Table 2). Validation included genebank staff taking pictures of the species in question, to subsequently determine actual species names, as opposed to just the names used by farmers in their local language.

#### 2.2.2. Conservation Tender 

Farmers participating in the FGDs also participated in a conservation tender training exercises and were then invited to later submit PACS conservation tender bids if they would be interested in participating as a group in activities to promote the conservation and sustainable use of CWR species. District Agricultural Crops Officers, Agriculture Extension Development Officers and Agricultural Extension Development Coordinators facilitated the formation of the farmers’ groups (in some cases this involved existing groups) in the target EPAs.

The tender process included the following steps:

- Explanation of what a conservation tender is and why it is being applied to the conservation of CWRs.
- Description of the way this tender for CWR conservation would operate. In this context:
  - Communities were informed that participation in the tender was voluntary and that they were at liberty to participate or not. They were also informed that the tender covered all CWRs found within their communities, although priority would be given to threatened CWRs as identified by previous scientific studies. (However, based on the samples community members provided and transect walk verification, it turned out that almost all of the species identified by the communities had in fact been previously targeted for conservation, hence there was no need to prioritise between them.) All community members were eligible to participate.
  - Farmers were advised that only the “best” offers, i.e., those with the highest benefits and lowest costs, would be selected. Social equity considerations—such as participation of vulnerable groups such as women, youth, and the poor—would nonetheless be taken into account as part of this process of identifying “best” offers. Communities would be selected up to the point where a certain total area of CWR has been conserved (or that the conservation budget has been fully used)
  - In-kind rewards, as defined be the farming groups themselves (e.g., tools, farm inputs, construction materials, etc.), would be paid only upon successful completion of the contracts and be awarded following verification visits.
- Discussion regarding different potential area management options (AMOs) were realised to give the communities an opportunity to select the AMOs most appropriate for their context and consider the potential cost of associated activities. Potential AMOs included the establishment of community conservation areas, management of crop field borders, and backyard conservation.
- Choice of type of in-kind reward/support the community would require to be able to participate in the provision of this public good conservation service. Training in how to complete the tender bid offer sheets was also provided and dates for their completion were agreed upon.

#### 2.2.3. Post-Tender Monitoring

Follow-up field visits were conducted to monitor the implementation of the action plans developed by farmers. During these monitoring sessions, farmers or groups of farmers were asked to report on what they had completed so far. Genebank staff randomly selected farmers from the group to explain at least one task that they had planned and how it was implemented. These reports were followed by triangulating responses from other group members and AEDOs. Genebank staff verified tasks undertaking/completion by actual site visits to locations where farmers had indicated they had mapped CWR occurrence in their community and requested farmers to bring CWR seed samples where they had indicated that they had collected seeds. This worked particularly well in Nkhotakota and Salima where a group of farmers brought seed samples of *Oryza longistaminata* that they had collected from their fields. The AEDO also supported monitoring during their regular visits to the sites where conservation activities were being undertaken. They were able to report monthly to the genebank staff including through spoken and written communications, as well as photo documentation of tasks being carried out.

## 3. Results and Discussion

### 3.1. Focus Group Discussion (FGD) Results

FGD participants preferred not to be grouped by gender but rather to participate in mixed groups. In total, 287 people participated in the 13 FGDs, of which approximately 69% were female and 29% were youths/younger farmers. Men considered that women were likely to have more knowledge about plants than men, since the women are the ones responsible for obtaining food and firewood in wild areas. By contrast, women considered that since men are responsible for obtaining timber and that some of them are traditional healers, men would have more knowledge about certain types of plants compared to them. 

In terms of CWR knowledge, across all sites the number of CWRs occurring in the communities was, with the exception of Salima, greater than expected relative to accessions and information held by the national genebank. Nkhotakota reported the highest number of CWRs relative to the other two sites. Presence at the sites of the CWRs was verified by genebank staff during transect walks (see Table 2). A large majority (75%) of farmers was aware of the existence of CWR and were able to relate these to the crops they cultivate, with women participating very actively and demonstrating a higher level of knowledge regarding the presence of CWR in their communities than men. Community knowledge regarding CWRs was also in evidence from the samples participants brought in of the CWRs occurring in their communities and their explanations regarding their use.

In terms of areas of occurrence, most farmers (87%) indicated that CWRs could be found in cultivated lands, especially in *dambo* lands (i.e., permanent or seasonal wetlands in valleys, depressions, or flood plains [33]) where the soils are fertile. Participants considered that most CWRs are declining across the sites because of expansion in agricultural production (leading to both habitat loss and loss due to selective weeding) and unsustainable harvesting. 

In terms of use, farmers reported that most of the CWRs were used for food and medicinal purposes. For example, wild *Vigna* species (a close relative of *Vigna unguiculata*) and most *Cucumis* species are consumed as a vegetable, while most *Solanum* species were used for medicinal purposes. Other species were also mentioned and verified during a transect walk. These include species in the genera *Ipomoea*, *Sorghum*, *Oryza*, *Prunus, and Dioscorea*, among others. 

As part of each FGD and based on the interest that the farmers showed in participating in CWR conservation activities within their communities, each group elaborated an action plan where they outlined specific activities to be undertaken and their timeframe. The work plan included identification of main tasks, task leaders, labour and timing requirements, locations where the group would meet to undertake the task and any tools/materials required. 

### 3.2. Competitive Tender Results

The 13 tender bid offers received revealed that all the groups (average size = 18) were interested in participating in conservation activities. A combination of AMOs were selected by farmers based on the type of the species and state of occurrence. Examples of AMOs proposed by farmers for *Oryza* included transplantation of any found in farmers’ fields to a designated conservation area within one of the group member’s fields or into rice field borders (verifiable indicators: number of CWR individuals, size of conservation area, number of participating farmers). For the *Sorghum* species found along the roadside, farmers proposed to collect seeds and plant them in a designated community conservation area (verifiable indicator: quantity of seeds collected). For those CWRs found in forest reserves, farmers agreed to prepare firebreaks, to avoid unsustainable harvesting, deforestation, or damaging it when collecting firewood (verifiable indicator: number of CWR individuals). Groups expected the tasks involved to require approximately 3.5 person hours a day over seven days during a month (equivalent to 36.75 working days over the year).

Bid offers averaged just over Malawian Kwacha (MWK) 20,000 (≈USD 25) p.a., which assuming no other benefits from participation would imply a shadow wage rate of USD 0.68 per day (USD 25/36.75), suggesting the opportunity costs of participation are significantly lower in these rural areas than that implied by the average per capita income level of USD 1.74. All groups selected their in-kind rewards to be paid in the form of farm tools/implements (such as hoes, watering cans, panga knives, and slashers) and stating that the items would be used communally by the group. The bid offers also revealed that the farmer groups preferred to implement CWR conservation activities on communal lands rather than on/around their individual plots. Two main reasons were given for this. Firstly, to publicise their conservation work, with a view to raising awareness regarding the existence and importance of such work; and, secondly, as not many farmers had rice fields where they could conserve Oryza species. Given that most CWRs are found on communal lands, a communal land approach makes it easier to conserve them in their natural environments. Furthermore, from the project’s perspective, such a communal land approach was also considered desirable, with a view to avoiding any suggestion of individual favouritism and helping to ensure that benefits were more widely/fairly shared. However, where priority CWRs were found on/around individual plots, it was agreed that the group as a whole would go there to verify this and discuss how best to manage it. Given the relatively low total cost of the 13 bid offers (MWK 260,500, equivalent to ≈USD 325) p.a., it was possible to select all the offers and develop CWR conservation action plans with all 13 groups. Although plot sizes varied, on average they measured 0.10 ha., thus totalling 1.3 ha, implying a cost of USD 250/ha (325/1.3). This is somewhat more expensive but of similar magnitude to that identified in Zambia of USD 23-91/ha [23].

### 3.3. Post-Tender Results

Post-tender monitoring and verification visits results revealed that, of the 13 groups, three (23.1%) fully complied with their action plans, as well as carried out additional tasks, for which they were duly rewarded extra; and a further eight (61.5%) fully complied with their action plan agreements and were rewarded in accordance with their bid offers. By contrast, only two groups (15.4%) had not fully completed their action plans and in these cases only partial payment was made. In the case of Tembwe EPA, Magaga Section (Salima), group membership was found to have declined to 25% out of the original group of 20 farmers.

Overall, the CWR-focussed PACS application revealed a high willingness amongst community groups to participate in conservation activities. As such, there appears to be significant potential for community engagement in CWR conservation activities that could complement that required in protected areas. However, despite the underlying cost-effectiveness of PACS schemes based on conservation tenders and the relatively low costs of CWR conservation implementation identified in this Malawi case study, the sustainability issues associated with the longer-term implementation of such incentive mechanisms need to be considered. As is the case with many natural resource-based payments for ecosystem services schemes, support payments need to be made periodically for conservation activities to continue to be undertaken. Furthermore, the fact that CWR are threatened globally will require CWR PACS schemes to be implemented on a much broader scale, which in addition to funding will require significant national implementing agency and farm community capacity-building. 

## 4. Conclusions

As in other countries, a significant proportion (27%) of Malawi’s CWR taxa are found outside of protected areas. A large majority (75%) of farmers was aware of the existence of CWR and were able to relate these to the crops they cultivate, with women demonstrating a higher level of knowledge regarding the presence of CWR in their communities than men. Farmers noted that CWR could mostly be found in cultivated lands where the soils are fertile and where they were used for food and medicinal purposes. Reasons for the declining presence of CWR across the sites were attributed to land clearing of unprotected areas for increased agricultural production and, in cases where there is direct use as a wild harvested crop or for medicinal purposes, unsustainable harvesting in their habitats (both wild and on farm). 

The CWR-focussed payments for agrobiodiversity conservation services application across 13 community groups in three districts of Malawi revealed a high willingness to participate in conservation activities, with average conservation tender bids per community group being a modest MWK 20,000 (USD 25) p.a. and covering 22 species of CWR associated with 17 different crops. As such, there appears to be significant potential for community engagement in CWR conservation activities that is urgently required to complement that required in Protected Areas and that this can be achieved at relatively low cost where appropriate incentive mechanisms can be implemented. 

While this payment was a one-off under the project, we interpret these as being necessary on an annual basis in order to ensure that CWR conservation activities are carried out over longer time scales. Further research/post-project monitoring would be required to determine the “persistence” of CWR conservation activities in the absence of further direct support and thus the actual need for repeated annual interventions. For PACS landraces in Peru, it has been shown that annual reinterventions are not necessarily required; however, landraces may be expected to have higher direct use values than CWR to motivate such persistence [21]. 

To address the global challenge of CWR loss, such approaches urgently need to be scaled up (along with identification of the funding sources required to implement such incentive mechanisms over the long-term) both within Malawi and other countries with CWRs that are a priority for conservation and found outside of protected areas. Further research regarding the costs of CWR conservation activities within protected areas is also required to fully cost national and global in situ CWR conservation activities; as well as to understand relative cost differences between protected area and community-based PACS approaches in cases at the margin where these might be substitutes rather than complementary.

Establishing pilot contracts with communities to conserve CWR was a first step. The second in the case of Malawi is to develop a post-PACS intervention strategy to facilitate direct access to the conserved CWR genetic resources by potential beneficiaries (i.e., breeders). How this goal is achieved is less well established in in situ conserved diversity compared to its routine application in ex situ genebanks. Therefore, the growing consensus, originally proposed by Maxted et al. (2016) and further described by Maxted (2021), is to provide user access to in situ conserved CWR diversity via the genebank. A backup sample is obtained from the in situ conserved CWR population and then treated as an ex situ sample in the genebank. However, genebank processing should exclude the most expensive element of ex situ storage, i.e., periodic population regeneration to maintain germination levels. Instead, when the seed viability of the in situ sample stored ex situ falls below 70–85%, a fresh sample is to be obtained from the host in situ population [34,35]. In such a context, the Malawi Plant Genetic Resource Centre has a key role to play in providing continued support for community CWR management, undertaking collection missions in such areas, storing, documenting, characterising, and having materials refreshed, and undertaking pre-breeding activities.

## Figures and Tables

**Table 1:** Workplan example for CWR conservation activities applied in Salima District.

Activity	Responsible Persons	Date	Location
Election of group leaders	All group members	22 March 2021	Lifidzi Value Addition Centre
Agreement regarding group participation rules	All group members	25 March 2021	Lifidzi Value Addition Centre
Mapping of CWR distribution	All group members	1 April 2021	Lifidzi Value Addition Centre
Informing community leaders about the CWR conservation initiative	Group leaders	31 March 2021	Chief’s house
Securing plot to conserve CWR	All group members	8 April 2021	Chief’s house
Land clearing	All group members	15 April 2021	Field
Land tilling	All group members	22 April 2021	Field
Preparing of planting beds	All group members	29 April 2021	Field
Collecting and planting of CWR seeds	All group members	6 May 2021	Field
CWR plot management	All group members	1 January 2022–1 December 2022	Field
Reporting of group progress	Group leaders	Monthly	Lifidzi Value Addition Centre
Post-project sustainability training	Genebank staff	18 February 2022	Lifidzi Value Addition Centre
Reward handover ceremony	Genebank staff	1 March 2022	Lifidzi Value Addition Centre

**Table 2:** Presence of CWRs identified by farmers in Nkhatabay, Nkhotakota, and Salima Districts.

Crop	Crop Wild Relatives	Nkhatabay District	Nkhotakota District	Nkhotakota District	Salima District	Salima District
		Chitheka EPA	Mphonde EPA	Linga EPA	Chipoka EPA	Tembwe EPA
Apple	*Prunus africana* (Hook. f.) Kalkman	X	-	-	-	-
2. Cowpeas	*Vigna unguiculata* L. (Walp.) subsp. *unguiculata* (wild populations)	X	X	X	X	X
3. Cucumbers	*Cucumis metuliferus* E. Mey. ex Naudin, *C. anguria* L., *C. shirsutus* Sond., C. *maderaspatanus* L.	X	X	X	X	X
4. Eggplants	*Solanum anguivi* Lam.	X	X	X	X	X
5. Ginger (Twisted)	*Costus afer* Ker Gawl.	X	X	X	-	-
6. Grapes	*Vitis* sp.	X	-	X	-	-
7. Green grams	*Vigna frutescens* A. Rich.	X	X	X	-	-
8. Guavas	*Psidium cattleyanum* Sabine	X	X	X	-	-
9. Mustard	*Brassica juncea* (L.) Czern.	-	-	X	-	-
10. Rice	*Oryza longistaminata* A. Chev. & Roehr.	X	X	X	X	X
11. Sesame	*Sesamum angustifolium* (Oliv.) Engl.	X	-	-	X	-
12. Sorghum	*Sorghum arundinaceum* (Desv.) Stapf	-	X	X	X	X
13. Sugarcane	*Saccharum* sp.	-	X	X	-	-
14. Sweet potato	*Ipomoea pileata* Roxb., *I. aquatica* Forssk.	X	X	X	X	X
15. Turmeric	*Curcuma* sp.	-	X	X	-	-
16. Yams (air yams)	*Dioscorea praehensilis* Benth.	X	X	-	X	-
17. Yams (ground yams)	*Dioscorea schimperiana* Hochst. ex Kunth	X	X	X	X	X
**Total Presence Verified**		13	13	14	9	7

X = verified presence of CWRs during transect walk; - = absence of CWRs or not observed during transect walk. Source: project survey.

## Data Availability

The data presented in this study are fully available within this article under Table 2 and Section 3.

## Appendix A

**Table A1:** Crop wild relatives identified by farmers in Malawi across Khaki Mponya et al. sites.

*Chenopodium ambrosioides*
*Coffea ligustroides*
*Coffea mufindiensis* subsp. *australis*
*Cucumis anguria* var. *anguria*
*Eleusine indica*
*Eleusine coracana* subsp. *africana*
*Ipomoea pileata*
*Ipomoea obscura* subsp. *obscura*
*Ipomoea tenuirostris*
*Oryza barthii*
*Oryza longistaminata*
*Prunus africana*
*Solanum aculeatissimum*
*Solanum anguivi*
*Solanum campylacanthum*
*Solanum hispidum*
*Solanum incanum*
*Solanum nigrum*
*Solanum panduriforme*
*Solanum richardii* subsp. *richardii*
*Solanum richardii*
*Solanum schumannianum*
*Solanum tarderemotum*
*Solanum terminale*
*Solanum terminale* subsp. *terminale*
*Solanum torvum*
*Sorghum bicolor* subsp. *arundinaceum*
*Sorghum versicolor*
*Vigna frutescens*
*Vigna unguiculata* var. *unguiculata*
*Vigna pygmaea*
*Vigna platyloba*
*Vigna oblongifolia*
*Vigna phoenix*
*Vigna heterophylla* subsp. *ambacensis*
*Vigna unguiculata* subsp. *dekindtiana*
*Vigna unguiculata* subsp. *spontanea*
*Vigna gazensis*
*Vigna vexillata* subsp. *angustifolia*
*Vigna vexillata* var. *vexillata*
*Vigna comosa*
*Vigna luteola*
*Vigna racemosa*
*Vigna reticulata*

Source: [32].
